# Supplementary material for: In-depth characterization of a selection of gut commensal bacteria reveals their functional capacities to metabolize dietary carbohydrates with prebiotic potential
Source: mSystems. 2024 Mar 5;9(4):e01401-23. doi: 10.1128/msystems.01401-23 (PMC11019791; doi:10.1128/msystems.01401-23)
Supplement: Text S1 — Supplemental methods. [file msystems.01401-23-s0002.docx]

**Supplemental Methods**

*Culture media*

A low nutrient culture medium (LNCM) was adapted from YCFA medium [1] as follows per 100 mL: 1.0 g Bacto^TM^ Tryptone (BD), 0. 25 g Bacto^TM^ Yeast Extract (BD), 0.4 g NaHCO3 (Prolabo), 0.045 g K2HPO4 (Prolabo), 0.045 g KH2PO4 (Merck), 0.09 g NaCl (Merck), 0.009 g MgSO4.7H2O (Prolabo), 0.009 g CaCl2.2H2O (Fluka), 0.1 mg resazurin (Sigma-Aldrich), 0.001 g hemin (Sigma-Aldrich) and 0.1 g cysteine (Sigma-Aldrich). The LNCM was supplemented with a filter-sterilised solution of vitamins (1 μg biotin, 1 μg cobalamin, 3 μg p-aminobenzoic acid, 5 μg folic acid, 15 μg pyridoxamine, 5 μg thiamine and 5 μg riboflavin), all purchased from Sigma-Aldrich. For the experiments carried out with *A. muciniphila*, the LNCM was enriched in 1% bovine mucin (Sigma-Aldrich). BHI medium was supplemented with 10 mg/L hemin, 5 mg/mL vitamin K1, 0.5% L-cysteine, 1% cellobiose and 1% maltose, all purchased from Sigma-Aldrich (France).

*SCFA analysis*

Chromatographic separation was achieved using a Fused Silica capillary column (15 m x 0.53 mm x 0.5 µm, film thickness) from Supelco (Sigma-Aldrich, France). Bacterial samples were centrifuged at 12,000 *g* for 15 min at 4°C, acidified overnight with 1:11 (v/v) 0.85 M phosphotungstic acid (Sigma-Aldrich, France) and 0.3 µL were injected in splitless mode (splitless time 3 min). The constant flow rate of H_2_ carrier gas was 10 mL/min and the inlet, column and FID detector temperatures were 200, 100 and 240°C, respectively. The oven was initially set at 100°C for 10 min, programmed at a rate of 20°C/min at 180 C and maintained for 2 min. Calibration curves were obtained for each SCFA using a standard mixture at a final concentration of 4 mM. The supernatants and calibration SCFA standard were spiked with 1:5 (v/v) internal standard solution of 2-ethyl butyric acid at a 20 mM final concentration in the samples. Phosphotungstic acid, 2ethyl butyric acid and volatile free acid mix were purchased from Sigma-Aldrich (France). All samples were analysed in duplicates, without any previous SCFA derivatisation. Data were integrated using OpenLab Chem station software (Agilent). The ratio between each SCFA peak area and the internal standard peak areas was plotted against standard concentrations to calculate the quantities for each analyte.

*Metabolite identification and annotation*

Liquid chromatography coupled to high-resolution mass spectrometry (LC-MS) analyses were performed using a U3000 liquid chromatography system coupled to an Exactive mass spectrometer (Thermo Fisher Scientific, France) fitted with an electrospray source operated in the positive and negative ion modes. Chromatographic separation was performed on a hypersil GOLD C18 1.9 μm, 2.1 mm × 150 mm column (C18) at 30°C (Thermo Fisher Scientific, France) and on a Sequant ZICpHILIC 5 µm, 2.1 mm x 150 mm column (HILIC) at 15°C (Merck, Germany). The combinaison of these systems allowed the cataloguing of as many classes of small chemical compounds as possible (hydrophobic, hydrophilic, negatively charged, and positively charged). Chromatographic systems were equipped with an on-line prefilter (Thermo Fisher Scientific, France). The experimental settings for each LC/MS condition are described in Boudah et al., 2014 [2]. Metabolite extraction was performed twice (for HILIC and C18 analyses) from 50 µL of supernatants after precipitation of proteins assisted by methanol, as previously described in Boudah et al., 2014 [2]. Raw data were manually inspected using Xcalibur version 2.1 (Thermo Fisher Scientific, France). Automatic peak detection and integration were performed using XCMS software [3]. XCMS features were filtered according to: *(i)* the correlation between the dilution factors of the QC samples and the areas of the chromatographic peaks (correlation coefficients greater than 0.7 to account for metabolites occurring at low concentrations and which are no longer detected in the most diluted samples), *(ii)* the repeatability (coefficients of variation obtained for the chromatographic peak areas of the QC samples should be less than 30 %) and *(iii)* the ratio of the chromatographic peak areas of the biological to blank samples above a value of 3. Optionally, if necessary, the chromatographic peak areas of each variable present in the XCMS peak lists were normalized using the LOESS algorithm in order to remove analytical drift induced by clogging of the ESI source observed in the course of analytical runs. Features were annotated by matching their m/z ± 10 ppm (quantity formed by dividing the ratio of the mass of an ion to the unified atomic mass unit, by its charge number) and retention time with referent molecules contained in intern [2] and public (KEGG, METLIN and HMDB) databases [4-6].

*Transcriptomic analyses*

Total RNA were isolated using RNeasy Mini Kit (Qiagen). Quality was verified on Agilent Bioanalyzer 2100 using RNA 6000 Pico Kit (Agilent Technologies). Directional RNA-Seq Libraries were constructed using the TruSeq Stranded Total RNA Library Prep Kit with bacteria Ribo-Zero reagents (Illumina). Libraries quality was assessed on Agilent Bioanalyzer 2100 using Agilent High Sensitivity DNA Kit (Agilent Technologies). Libraries were pooled in equimolar proportions and sequenced on a single read 75 pb run. Demultiplexing was performed with bcl2fastq2 v2.18.12. Adapters were trimmed with Cutadapt v1.15. Reads (> 10 pb) were mapped on high-quality genomes (Table 1) and counted using subread featureCounts v1.5.2. Gene annotations were obtained from the NCBI database of bacterial genomes and completed with automated CAZyme-encoding genes using dbCAN2 v9 annotation tool (http://cys.bios.niu.edu/dbCAN2) combining HMMER search against the dbCAN hidden Markov model (HMM) database and DIAMOND search against the CAZy database [7]. Results were filtered to exclude all matches with an e-value threshold of 1e-102 and an alignment coverage threshold of 0.35. Samples with low depth sequencing (less than 10^7^ total read counts) were removed. Low count genes across all libraries were filtered based on count per million greater than 1 in at least 2 or 3 samples corresponding to the minimum number of biological replicates. Normalisation was performed based on the depth of libraries and the distribution of reads per sample, as discussed in Dillies et al. [8]. Trimmed mean of M values (TMM) normalisation was performed using the ‘calcNormFactors’ function from edgeR package v3.38.2 [9]. To explore the relationships between genes and biological functions, gene ontology (GO) annotations encompassing biological process were employed using *ViSEAGO* v1.10. Functional enrichment analysis identified over-represented GO terms among differentially expressed genes at a significant level of 0.1 using the ‘classic’ algorithm with ‘fisher’ test. A minimal number of genes annotated to a GO term was settled at 5 using ‘genes_nodeSize’ (See the Table below).


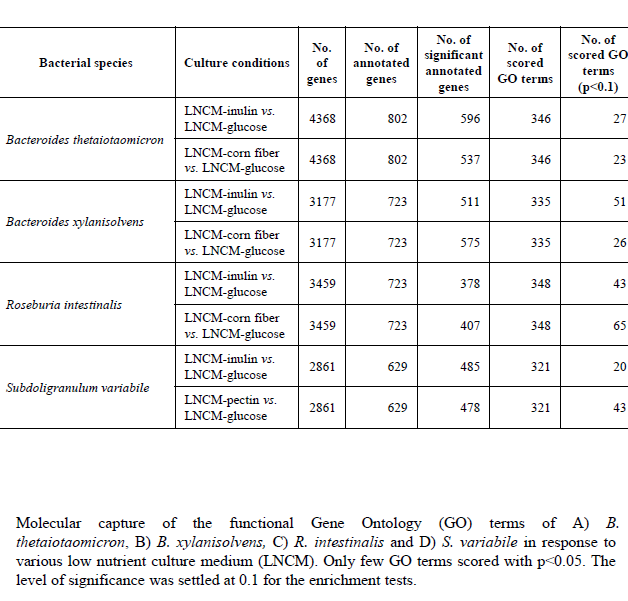


*References*

1. Duncan, S.H., et al., *The role of pH in determining the species composition of the human colonic microbiota.* Environ Microbiol, 2009. **11**(8): p. 2112-22.

2. Boudah, S., et al., *Annotation of the human serum metabolome by coupling three liquid chromatography methods to high-resolution mass spectrometry.* J Chromatogr B Analyt Technol Biomed Life Sci, 2014. **966**: p. 34-47.

3. Giacomoni, F., et al., *Workflow4Metabolomics: a collaborative research infrastructure for computational metabolomics.* Bioinformatics, 2015. **31**(9): p. 1493-5.

4. Kanehisa, M. and S. Goto, *KEGG: Kyoto Encyclopedia of Genes and Genomes.* Nucleic Acids Research, 2000. **28**(1): p. 27-30.

5. Smith, C.A., et al., *METLIN: a metabolite mass spectral database.* Ther Drug Monit, 2005. **27**(6): p. 747-51.

6. Wishart, D.S., et al., *HMDB: the Human Metabolome Database.* Nucleic Acids Res, 2007. **35**(Database issue): p. D521-6.

7. Dillies, M.A., et al., *A comprehensive evaluation of normalization methods for Illumina high-throughput RNA sequencing data analysis.* Brief Bioinform, 2013. **14**(6): p. 671-83.

8. Robinson, M.D., D.J. McCarthy, and G.K. Smyth, *edgeR: a Bioconductor package for differential expression analysis of digital gene expression data.* Bioinformatics, 2010. **26**(1): p. 139-40.

9. Zhang, H., et al., *dbCAN2: a meta server for automated carbohydrate-active enzyme annotation.* Nucleic Acids Res, 2018. **46**(W1): p. W95-w101.
