# Supplementary material for: In-depth characterization of a selection of gut commensal bacteria reveals their functional capacities to metabolize dietary carbohydrates with prebiotic potential
Source: mSystems. 2024 Mar 5;9(4):e01401-23. doi: 10.1128/msystems.01401-23 (PMC11019791; doi:10.1128/msystems.01401-23)
Supplement: Fig. S1 — Production of short-chain fatty acids (SCFA) after 24h-culture of commensal bacteria. [file msystems.01401-23-s0005.pdf]

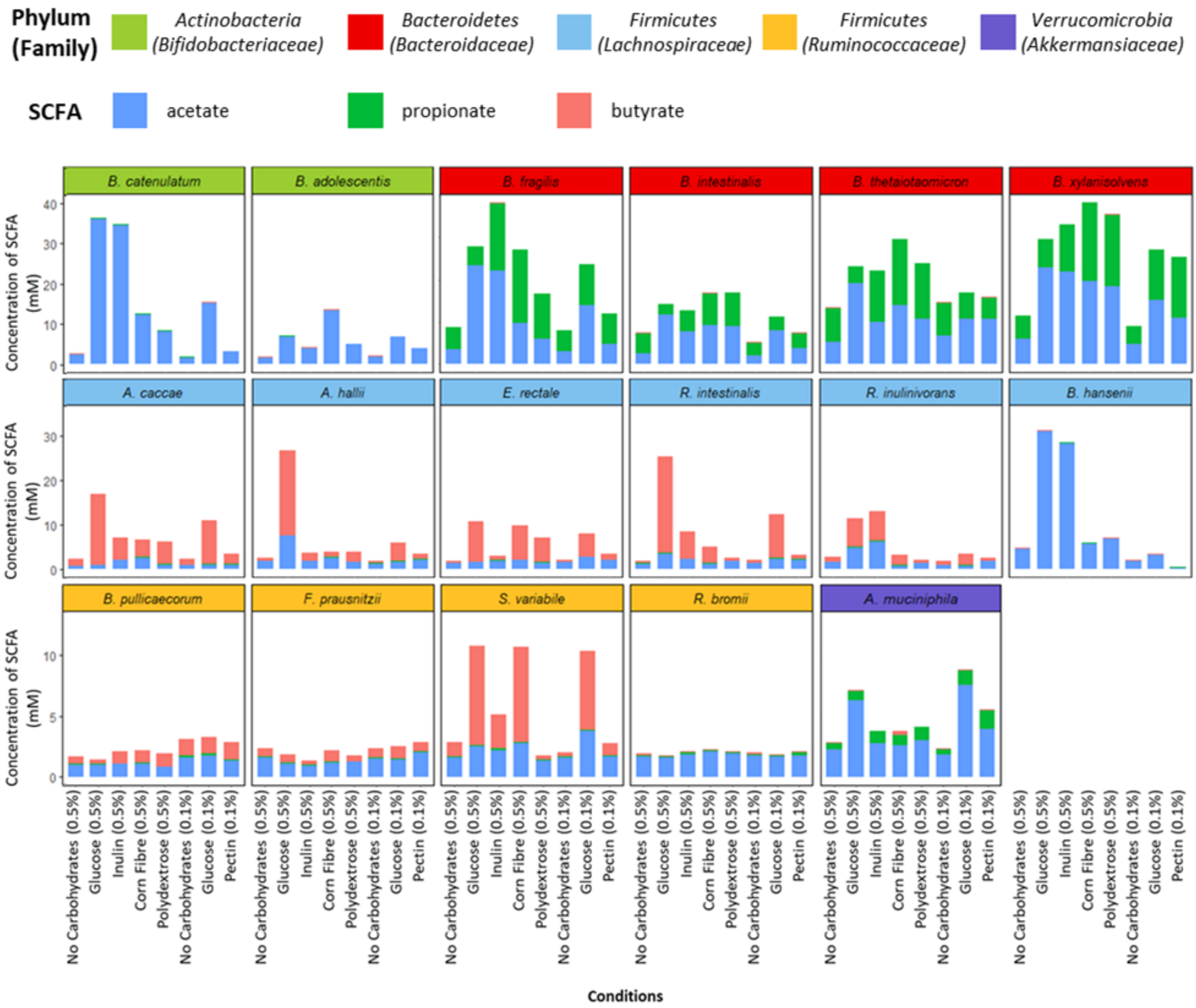

Figure S1: Production of short-chain fatty acids (SCFA) after 24h-culture of commensal bacteria with different carbon sources, including glucose, agave inulin, corn fiber, polydextrose, and citrus pectin. The SCFA concentrations are expressed in mM. The bar plots represent the overall mean of acetate, propionate and butyrate concentrations.
