# Supplementary material for: In-depth characterization of a selection of gut commensal bacteria reveals their functional capacities to metabolize dietary carbohydrates with prebiotic potential
Source: mSystems. 2024 Mar 5;9(4):e01401-23. doi: 10.1128/msystems.01401-23 (PMC11019791; doi:10.1128/msystems.01401-23)
Supplement: Fig. S2 — Global metabolomic profiles of six commensal bacteria. [file msystems.01401-23-s0006.pdf]

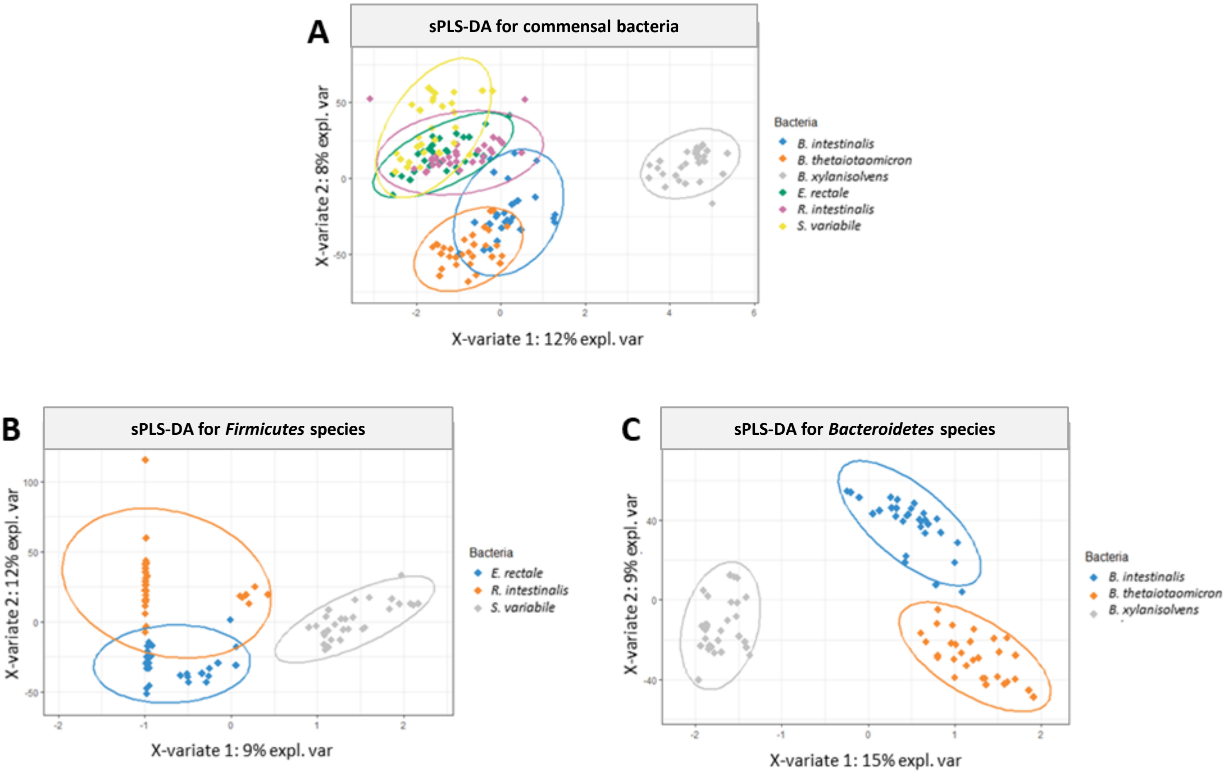

Figure S2: Global metabolomic profiles of six commensal bacteria compared within all bacteria (A), within *Firmicutes* phylum (B), and within *Bacteroidetes* phylum (C). Each condition was performed in six replicates, in addition to five replicates of the initial non-inoculated low nutrient culture media. Scatter plots of the first two sPLS-DA components were obtained. All ellipses were drawn assuming a multivariate t-distribution with a confidence level of 0.95.
