# Supplementary figures and images for: In-depth characterization of a selection of gut commensal bacteria reveals their functional capacities to metabolize dietary carbohydrates with prebiotic potential
Source: mSystems. 2024 Mar 5;9(4):e01401-23. doi: 10.1128/msystems.01401-23 (PMC11019791; doi:10.1128/msystems.01401-23)

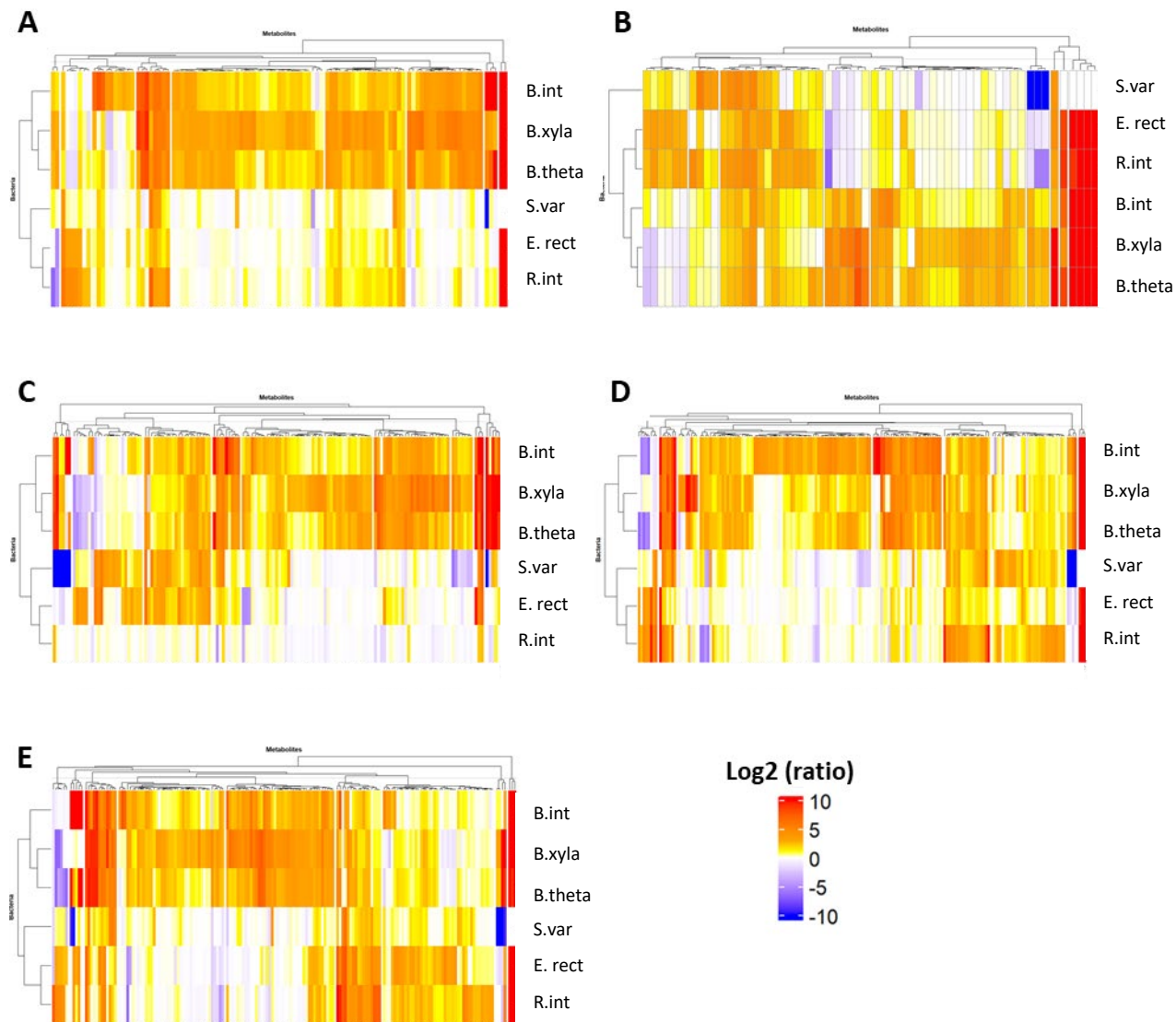

Supplement: Fig. S3 — Heatmaps of differentially abundant metabolites, annotated with public databases. [file msystems.01401-23-s0007.pdf]
