# Supplementary material for: In-depth characterization of a selection of gut commensal bacteria reveals their functional capacities to metabolize dietary carbohydrates with prebiotic potential
Source: mSystems. 2024 Mar 5;9(4):e01401-23. doi: 10.1128/msystems.01401-23 (PMC11019791; doi:10.1128/msystems.01401-23)
Supplement: Table S1 — Features of 17 selected commensal bacteria and genomic information. [file msystems.01401-23-s0009.pdf]

Phylum (Family)	Bacterial Species	International Collection	Strain Designation	RefSeq Assembly Accession	Assembly Level	Total Length (kb)	CDS	No. CAZy	Clinical Observations
Actinobacteria (Bifidobacteriaceae)	*Bifidobacterium adolescentis*	DSM 20083T	E194an (variant a)	GCF_000010425.1	complete	2.1	1676	71	Prevalent in the large intestine of healthy individuals (King et al. 2019)<br>Protective effect on the integrity of the intestinal barrier (Qi et al. 2023)<br>Abundance modified in IBS (Jeffery et al. 2011), CD (Gevers et al. 2014), and MetS (Haro et al. 2016)
	Bifidobacterium catenulatum	DSM 16992T	B669	GCF_000173455.1	contig	2.1	1677	74	Protective effect in allergic diseases and asthma (Ismail et al. 2016, Mahdavinia et al. 2023)<br>Abundance modified in IBS (Kerckhoffs et al. 2009)
Bacteroidetes (Bacteroidaceae)	*Bacteroides fragilis*	DSM 2151T	EN-2	GCF_000025985.1	complete	5.2	4544	239	Prevalent in the large intestine of healthy individuals (King et al. 2019)<br>Abundance modified in CRC (Wang et al. 2012)<br>Primary polysaccharides degrader (Zafar and Saier 2021)
	Bacteroides intestinalis	DSM 17393T	341	GCF_000172175.1	contig	6.1	4655	407	Primary polysaccharides degrader (Pereira et al. 2021)
	Bacteroides thetaiotaomicron	DSM 2079T	E50 (VPI-5482)	GCF_000011065.1	complete	6.3	4794	382	Prevalent in the large intestine of healthy individuals (King et al. 2019)<br>Abundance modified in MetS (Haro et al. 2016)<br>Primary polysaccharides degrader (Zafar and Saier 2021)
	Bacteroides xylanisolvens	DSM 18836T	XB1A	GCF_000273315.1	scaffold	6.1	4621	352	Prevalent in the large intestine of healthy individuals (King et al. 2019)<br>Primary polysaccharides degrader (Zafar and Saier 2021)<br>Strain identified as next generation probiotic (Brodmann et al. 2017)
Firmicutes (Lachnospiraceae)	*Eubacterium rectale*<br>*Syn. Agathobacter rectalis*	ATCC 33656T	VPI-0990	GCF_000020605.1	complete	3.4	3288	83	Prevalent in the large intestine of healthy individuals (Almeida et al. 2019, King et al. 2019)<br>Potential beneficial bacteria (Mukherjee et al. 2020)<br>Abundance modified in MetS (Haro et al. 2016, Qin et al. 2012, Jamar et al. 2018), IBS (Rajilić–Stojanović et al. 2011), and CD (Gevers et al. 2014)
	Anaerobutyricum hallii<br>*Syn. Eubacterium hallii*	DSM 17630	L2-7	GCF_900209925.1_EH1	complete	3.7	3076	35	Potential beneficial bacteria (Mukherjee et al. 2020)<br>Abundance modified in IBS (Jeffery et al. 2011)
	Anaerostipes caccae	DSM 14662T	L1-92	GCF_000154305.1	scaffold	3.6	3317	36	Protective effect in food allergy (Feehley et al. 2019)<br>Abundance modified in CRC (Montalban-Arques et al. 2021)
	Blautia hansenii	DSM 20583T	VPI-C7-24	GCF_002222595.2	complete	3.1	2974	55	Abundance modified in IBS (Rajilić–Stojanović et al. 2011), and CD (Gevers et al. 2014)<br>Potential beneficial bacteria (Liu et al. 2021)
	Roseburia intestinalis	DSM 14610T	L1-82	GCF_000156535.1	scaffold	4.4	4267	173	Prevalent in the large intestine of healthy individuals (King et al. 2019)<br>Abundance modified in CRC (Montalban-Arques et al. 2021), MetS (Qin et al. 2012), and CD (Gevers et al. 2014)<br>*Firmicutes* polysaccharides degraders (Tamanai-Shacoori et al. 2017)
	Roseburia inulinivorans	DSM 16841T	A2-194	GCF_000174195.1	contig	4.0	3926	58	Potential beneficial bacteria (Tamanai-Shacoori et al. 2017)<br>Abundance modified in MetS (Qin et al., 2012)
Firmicutes (Ruminococcaceae)	*Butyricicoccus pullicaecorum*	DSM 23266T	25-3	GCF_900167005.1	scaffold	3.3	3103	34	Protective effect in inflammation (Eeckhaut et al. 2013), and CRC (Chang et al. 2020)
	Faecalibacterium prausnitzii	DSM 17677	A2-165	GCF_002734145.1	complete	3.1	2956	60	Prevalent in the large intestine of healthy individuals (King et al. 2019)<br>Abundance modified in CRC (Montalban-Arques et al. 2021), CD (Gevers et al. 2014), and MetS (Haro et al. 2016)<br>Anti-inflammatory properties (Touch et al. 2022)
	Ruminococcus bromii	ATCC 27255	VPI 6883	GCF_002834225.1	contig	2.1	1998	38	Prevalent in the large intestine of healthy individuals (King et al. 2019, Almeida et al. 2019)<br>Abundance modified in food allergy (Bao et al. 2021)
	Subdoligranulum variabile	DSM 15176T	Bl 114	GCF_000157955.1	scaffold	3.2	3040	49	Protective effect in food allergy (Abdel-Gadir et al. 2019)
Verrucomicrobia (Akkermansiaceae)	*Akkermansia muciniphila*	ATCC BAA-835T	Muc	GCF_000020225.1	complete	2.7	2257	107	Prevalent in the large intestine of healthy individuals (King et al. 2019)<br>Abundance modified in MetS (Jamar et al. 2018, Cani et al. 2022)

Table S1: Features of 17 selected commensal bacteria and genomic information. All strains are human isolates except *Butyricoccus pullicaecorum*. T stands for “Type” bacterial strain. Draft or complete genomic data [RefSeq Assembly Accession, Assembly level, Total Length, number of coding sequences (CDS)] were obtained for each selected bacteria from the National Center for Biotechnology Information (NCBI) database (https://www.ncbi.nlm.nih.gov). The prediction of CAZymes of a genome was performed using the dbCAN2 v9 annotation tool (http://cys.bios.niu.edu/dbCAN2) (Zhang et al., 2018). As recommended by the database, the combination of the HMMER search against the dbCAN hidden Markov model (HMM) database and the DIAMOND search against the CAZy database allowed to significantly identify the CAZyme-encoding genes. The results were filtered to exclude all matches with an e-value threshold of 1e-102 and an alignment coverage threshold of 0.35. The selection of bacteria was guided by clinical observations. In comparison with healthy individuals, bacterial species with modified relative abundances in individuals suffering from food allergy, colorectal cancer (CRC), metabolic syndrome (MetS), irritable bowel syndrome (IBS) and Crohn disease (CD) were pinpointed as potential beneficial bacteria.

Abdel-Gadir, A., et al., *Microbiota therapy acts via a regulatory T cell MyD88/RORγt pathway to suppress food allergy.* Nature Medicine, 2019. **25**(7): p. 1164-1174.

Almeida, A., et al., *A new genomic blueprint of the human gut microbiota.* Nature, 2019. **568**(7753): p. 499-504.

Bao, R., et al., *Fecal microbiome and metabolome differ in healthy and food-allergic twins.* The Journal of Clinical Investigation, 2021. **131**(2).

Brodmann, T., et al., *Safety of Novel Microbes for Human Consumption: Practical Examples of Assessment in the European Union.* Frontiers in Microbiology, 2017. **8**.

Cani, P.D., et al., *Akkermansia muciniphila: paradigm for next-generation beneficial microorganisms.* Nature Reviews Gastroenterology & Hepatology, 2022. **19**(10): p. 625-637.

Chang, S.-C., et al., *A gut butyrate-producing bacterium Butyricicoccus pullicaecorum regulates short-chain fatty acid transporter and receptor to reduce the progression of 1,2-dimethylhydrazine-associated colorectal cancer.* Oncol Lett, 2020. **20**(6): p. 327.

Eeckhaut, V., et al., *Butyricicoccus pullicaecorum in inflammatory bowel disease.* Gut, 2013. **62**(12): p. 1745-52.

Feehley, T., et al., *Healthy infants harbor intestinal bacteria that protect against food allergy.* Nature Medicine, 2019. **25**(3): p. 448-453.

Gevers, D., et al., *The treatment-naive microbiome in new-onset Crohn's disease.* Cell Host Microbe, 2014. **15**(3): p. 382-392.

Haro, C., et al., *The gut microbial community in metabolic syndrome patients is modified by diet.* J Nutr Biochem, 2016. **27**: p. 27-31.

Ismail, I.H., et al., *Early gut colonization by Bifidobacterium breve and B. catenulatum differentially modulates eczema risk in children at high risk of developing allergic disease.* Pediatr Allergy Immunol, 2016. **27**(8): p. 838-846.

Jamar, G., et al., *Relationship between fatty acids intake and Clostridium coccoides in obese individuals with metabolic syndrome.* Food Research International, 2018. **113**: p. 86-92.

Jeffery, I.B., et al., *An irritable bowel syndrome subtype defined by species-specific alterations in faecal microbiota.* Gut, 2012. **61**(7): p. 997-1006.

Kerckhoffs, A.P., et al., *Lower Bifidobacteria counts in both duodenal mucosa-associated and fecal microbiota in irritable bowel syndrome patients.* World journal of gastroenterology: WJG, 2009. **15**(23): p. 2887.

King, C.H., et al., *Baseline human gut microbiota profile in healthy people and standard reporting template.* PLoS One, 2019. **14**(9): p. e0206484.

Liu, X., et al., *Blautia-a new functional genus with potential probiotic properties?* Gut Microbes, 2021. **13**(1): p. 1-21.

Mahdavinia, M., et al., *Gut microbiome is associated with asthma and race in children with food allergy.* J Allergy Clin Immunol, 2023.

Montalban-Arques, A., et al., *Commensal Clostridiales strains mediate effective anti-cancer immune response against solid tumors.* Cell Host Microbe, 2021. **29**(10): p. 1573-1588.e7.

Mukherjee, A., et al., *Gut microbes from the phylogenetically diverse genus Eubacterium and their various contributions to gut health.* Gut Microbes, 2020. **12**(1): p. 1802866.

Pereira, G.V., et al., *Degradation of complex arabinoxylans by human colonic Bacteroidetes.* Nature Communications, 2021. **12**(1): p. 459.

Qi, Y., et al., *Heat-inactivated Bifidobacterium adolescentis ameliorates colon senescence through Paneth-like-cell-mediated stem cell activation.* Nat Commun, 2023. **14**(1): p. 6121.

Qin, J., et al., *A metagenome-wide association study of gut microbiota in type 2 diabetes.* Nature, 2012. **490**(7418): p. 55-60.

Rajilić–Stojanović, M., et al., *Global and Deep Molecular Analysis of Microbiota Signatures in Fecal Samples From Patients With Irritable Bowel Syndrome.* Gastroenterology, 2011. **141**(5): p. 1792-1801.

Tamanai-Shacoori, Z., et al., *Roseburia spp.: a marker of health?* Future Microbiol, 2017. **12**: p. 157-170.

Touch, S., et al., *Human CD4+CD8α+ Tregs induced by Faecalibacterium prausnitzii protect against intestinal inflammation.* JCI Insight, 2022. **7**(12).

Wang, T., et al., *Structural segregation of gut microbiota between colorectal cancer patients and healthy volunteers.* The ISME Journal, 2012. **6**(2): p. 320-329.

Zafar, H. and M.H. Saier, Jr., *Gut Bacteroides species in health and disease.* Gut Microbes, 2021. **13**(1): p. 1-20.

Zhang, H., et al., *dbCAN2: a meta server for automated carbohydrate-active enzyme annotation.* Nucleic Acids Res, 2018. **46**(W1): p. W95-w101.
