# Supplementary material for: In-depth characterization of a selection of gut commensal bacteria reveals their functional capacities to metabolize dietary carbohydrates with prebiotic potential
Source: mSystems. 2024 Mar 5;9(4):e01401-23. doi: 10.1128/msystems.01401-23 (PMC11019791; doi:10.1128/msystems.01401-23)
Supplement: Table S2 — Statistical description of the four fermentation clusters. [file msystems.01401-23-s0010.pdf]

A

	p-value	DF
Bacteria	1.86e-103	48
Phylum	1.89e-95	12
Condition	6.18e-33	21

B

	p-value
Isovalerate	3.54e-242
Propionate	8.45e-147
Acetate	1.64e-135
Butyrate	9.99e-122
Isobutyrate	4.35e-105

D

		Mean in Cluster	Overall Mean	SD in Cluster	Overall SD	p-value
**Cluster N**	Butyrate	0.65	1.61	0.89	3.56	6.71e-78
	Isobutyrate	0.16	0.24	0.14	0.23	8.75e-26
	Acetate	2.92	5.44	3.19	7.18	2.73e-27
	Propionate	0.24	2.15	0.45	4.48	2.89e-39
	Isovalerate	0.04	0.41	0.13	0.84	3.41e-41
**Cluster G**	Butyrate	8.91	1.61	5.47	3.56	6.71e-78
	Acetate	2.77	5.44	2.87	7.18	7.08e-04
	Propionate	0.09	2.15	0.04	4.48	3.03e-05
	Isobutyrate	0.13	0.24	0.10	0.23	2.40e-05
	Isovalerate	0.02	0.41	0.05	0.84	2.26e-05
**Cluster A**	Isovalerate	2.14	0.41	0.64	0.84	1.17e-104
	Propionate	10.33	2.15	5.20	4.48	3.69e-82
	Isobutyrate	0.64	0.24	0.22	0.23	1.51e-71
	Acetate	9.20	5.44	5.53	7.18	3.90e-08
	Butyrate	0.08	1.61	0.03	3.56	5.84e-06
**Cluster G/I**	Acetate	26.61	5.44	7.41	7.18	2.05e-70
	Propionate	3.76	2.15	4.03	4.48	3.04e-02
	Butyrate	0.09	1.61	0.04	3.56	9.93e-03

C

		Cla/Mod	p-value
**Cluster N**	Phylum=*Firmicutes_Ruminococcaceae*	91	6.89e-15
	Phylum=*Verrucomicrobia*	100	2.97e-07
	Bacteria=*R.bromii*	100	2.97e-07
	Bacteria=*F.prausnitzii*	100	2.97e-07
	Bacteria=*B.pullicaecorum*	100	2.97e-07
	Bacteria=*B.adolescentis*	100	2.97e-07
	Bacteria=*A.muciniphila*	100	2.97e-07
	Phylum=*Actinobacteria*	88	7.56e-06
	Condition=Pectin_0.1%	82	3.76e-04
	Condition=No_Carbohydrates_0.1%	82	3.76e-04
	Condition=No_Carbohydrates_0.5%	76	1.68e-02
	Bacteria=*A.caccae*	47	4.95e-02
	Condition=Glucose_0.1%	50	1.48e-02
	Bacteria=*B.intestinalis*	38	2.31e-03
	Condition=Glucose_0.5%	38	6.25e-06
	Bacteria=*B.thetaiotaomicron*	19	1.26e-07
	Bacteria=*B.xylanisolvens*	0	1.61e-15
	Bacteria=*B.fragilis*	0	1.61e-15
	Phylum=*Bacteroidetes*	14	1.15e-40
**Cluster G**	Phylum=*Firmicutes_Lachnospiraceae*	31	2.53e-19
	Bacteria=*A.caccae*	53	2.50e-08
	Bacteria=*E.rectale*	50	2.23e-07
	Condition=Glucose_0.5%	32	1.01e-05
	Condition=Glucose_0.1%	32	1.01e-05
	Bacteria=*S.variabile*	38	3.64e-04
	Bacteria=*R.intestinalis*	38	3.64e-04
	Phylum=*Verrucomicrobia*	0	9.20e-03
	Bacteria=*R.bromii*	0	9.20e-03
	Bacteria=*F.prausnitzii*	0	9.20e-03
	Bacteria=*B.xylanisolvens*	0	9.20e-03
	Bacteria=*B.thetaiotaomicron*	0	9.20e-03
	Bacteria=*B.pullicaecorum*	0	9.20e-03
	Bacteria=*B.intestinalis*	0	9.20e-03
	Bacteria=*B.hansenii*	0	9.20e-03
	Bacteria=*B.fragilis*	0	9.20e-03
	Bacteria=*B.catenulatum*	0	9.20e-03
	Bacteria=*B.adolescentis*	0	9.20e-03
	Bacteria=*A.muciniphila*	0	9.20e-03
	Phylum=*Actinobacteria*	0	6.11e-05
	Condition=Pectin_0.1%	0	3.18e-05
	Condition=No_Carbohydrates_0.5%	0	3.18e-05
	Condition=No_Carbohydrates_0.1%	0	3.18e-05
	Phylum=*Bacteroidetes*	0	8.17e-10

		Cla/Mod	p-value
**Cluster A**	Phylum=*Bacteroidetes*	72	7.71e-75
	Bacteria=*B.fragilis*	81	1.33e-16
	Bacteria=*B.xylanisolvens*	75	6.97e-14
	Bacteria=*B.thetaiotaomicron*	69	1.81e-11
	Bacteria=*B.intestinalis*	63	2.54e-09
	Phylum=*Verrucomicrobia*	0	2.19e-03
	Bacteria=*S.variabile*	0	2.19e-03
	Bacteria=*R.inulinivorans*	0	2.19e-03
	Bacteria=*R.intestinalis*	0	2.19e-03
	Bacteria=*R.bromii*	0	2.19e-03
	Bacteria=*F.prausnitzii*	0	2.19e-03
	Bacteria=*E.rectale*	0	2.19e-03
	Bacteria=*B.pullicaecorum*	0	2.19e-03
	Bacteria=*B.hansenii*	0	2.19e-03
	Bacteria=*B.catenulatum*	0	2.19e-03
	Bacteria=*B.adolescentis*	0	2.19e-03
	Bacteria=*A.muciniphila*	0	2.19e-03
	Bacteria=*A.hallii*	0	2.19e-03
	Bacteria=*A.caccae*	0	2.19e-03
	Phylum=*Actinobacteria*	0	3.10e-06
	Condition=Glucose_0.5%	0	1.32e-06
	Phylum=*Firmicutes_Ruminococcaceae*	0	1.25e-12
	Phylum=*Firmicutes_Lachnospiraceae*	0	2.97e-20
**Cluster G/I**	Condition=Glucose_0.5%	29	2.26e-11
	Condition=Inulin_0.5%	21	1.36e-05
	Phylum=*Bacteroidetes*	14	1.42e-04
	Bacteria=*B.xylanisolvens*	25	4.12e-04
	Bacteria=*B.hansenii*	25	4.12e-04
	Bacteria=*B.catenulatum*	25	4.12e-04
	Bacteria=*B.fragilis*	19	1.27e-02
	Phylum=*Actinobacteria*	13	4.68e-02
	Condition=Polydextrose_0.5%	0	9.15e-03
	Condition=Pectin_0.1%	0	9.15e-03
	Condition=Glucose_0.1%	0	9.15e-03
	Condition=Corn_Fiber_0.5%	0	9.15e-03
	Condition=No_Carbohydrates_0.5%	0	9.15e-03
	Condition=No_Carbohydrates_0.1%	0	9.15e-03
	Phylum=*Firmicutes_Ruminococcaceae*	0	7.84e-05

Table S1: Statistical description of the four fermentation clusters. These results suggested functional hierarchisation of the metabolic interactions between the dietary carbohydrates and the commensal bacteria. (A) Statistical analysis of the clusters and the explicative variables that include bacteria, phyla and conditions. Chi^2 tests are performed. The explicative variables are classified according to their decreasing statistical significance. (B) Statistical analysis of the clusters and the quantitative variables that include acetate, propionate, isobutyrate, butyrate and isovalerate. ANOVA tests are performed on each quantitative variable with each cluster as the explicative variable. The quantitative variables are classified according to their decreasing statistical significance. (C) Description of each cluster based on the explicative variables. Each bacteria, phyla and conditions are represented in different proportions according to each group. (D) Description of each cluster based on the quantitative variables. The mean and standard deviation of each quantitative variables are reported for each cluster in comparison with the overall data set. T-Student tests are performed to compare the mean of each cluster and the mean of the overall data set.
