# Supplementary material for: In-depth characterization of a selection of gut commensal bacteria reveals their functional capacities to metabolize dietary carbohydrates with prebiotic potential
Source: mSystems. 2024 Mar 5;9(4):e01401-23. doi: 10.1128/msystems.01401-23 (PMC11019791; doi:10.1128/msystems.01401-23)
Supplement: Fig. S4 — Examples of biological functions expressed in 4 commensal bacteria. [file msystems.01401-23-s0008.pdf]

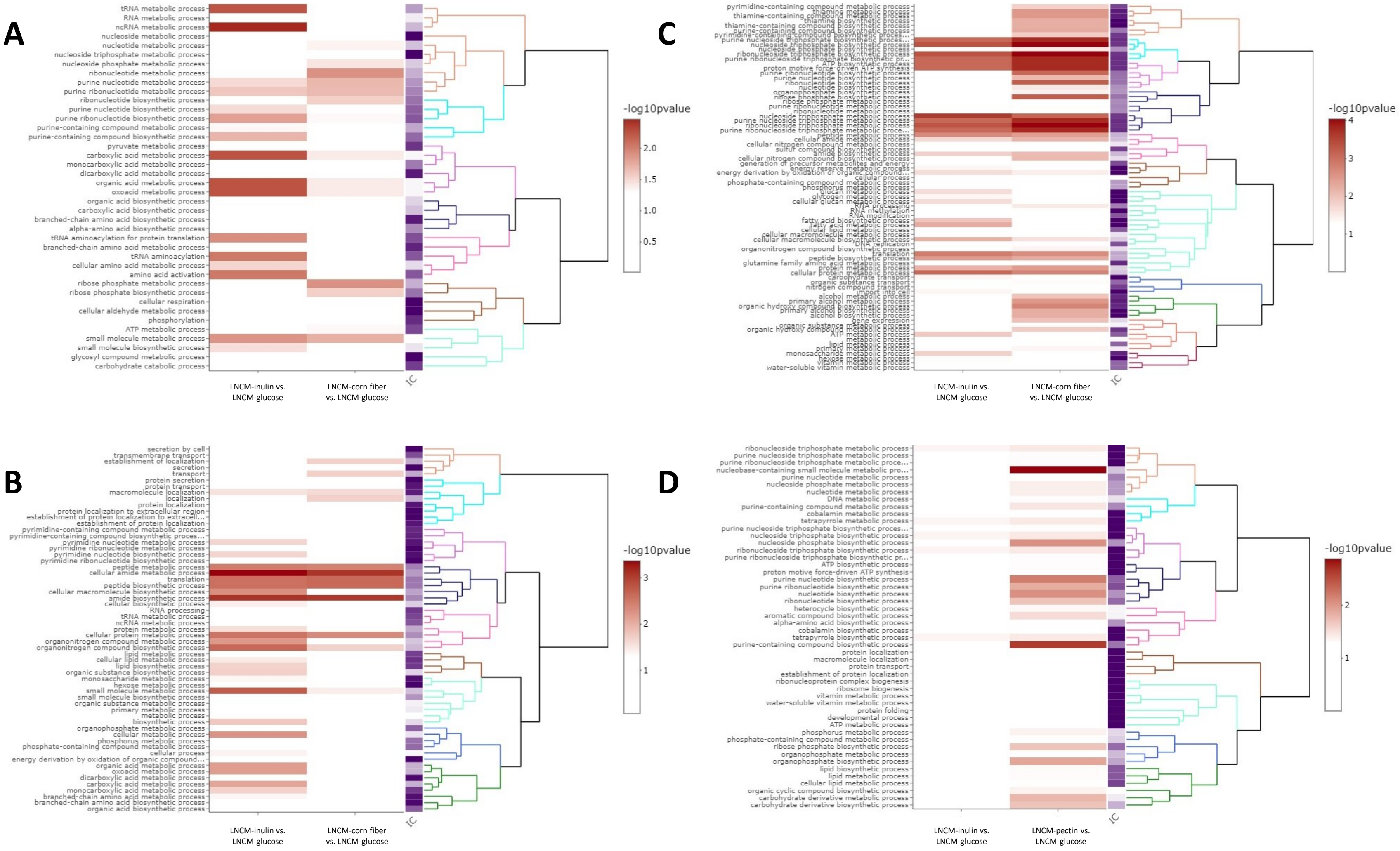

Figure S4: Examples of biological functions expressed in A) *B. thetaiotaomicron*, B) *B. xylanisolvens,* C) *R. intestinalis* and D) *S. variabile* in response to various carbon sources. Heatmaps of enriched Gene Ontology (GO) terms were grouped using a hierarchical clustering using Wang's semantic similarity and Ward aggregation criterion.
